# Cost-effectiveness of digital interventions for mental health: current evidence, common misconceptions, and future directions

**DOI:** 10.3389/fdgth.2024.1486728

**Published:** 2024-10-21

**Authors:** Claudia Buntrock

**Affiliations:** Faculty of Medicine, Institute of Social Medicine and Health Systems Research, Otto von Guericke University Magdeburg, Magdeburg, Germany

**Keywords:** cost-effectiveness, digital interventions, mental health, economic evaluations, current evidence

## Introduction

Digital interventions have been instrumental in addressing mental health problems for over a decade. These technology-based tools are designed to provide psychological support and facilitate therapeutic activities for individuals experiencing mental health challenges. They include Internet-based programs and mobile applications, among many other digital tools, that apparently promised an accessible, scaled, and economically efficient solution. In 2019, approximately 1 in 8 individuals, equating to 970 million people globally, were affected by a mental disorder, with anxiety and depressive disorders being the most prevalent (1). As mental health disorders continue to exert significant pressure on global health systems and economies, assessing the cost-effectiveness of these digital interventions becomes increasingly critical.

## History and importance of economic evaluations in healthcare

Economic evaluations address the fundamental economic problem of scarcity, where the demand for resources is greater than what is available (2). In healthcare, this will be balancing the choices between alternatives to maximize health outcomes in the face of a certain budget. This requires a rigorous analysis of the costs and benefits of different healthcare services, ensuring that resources are allocated efficiently and effectively. Economic evaluations in healthcare have evolved significantly since the 1960s (3). The development of quality-adjusted life years (QALYs) in the early 1970s represented a huge step forward in that it provided a single measure allowing comparisons of the relative effectiveness of different healthcare interventions (4, 5). The development of disability-adjusted life years (DALYs) by the World Bank and WHO in 1990 further refined this approach by incorporating both morbidity and mortality into the assessment (6). It was also during the 1990s that the major institutions in this regard, shaping the field of pharmacoeconomics, were set up - the International Society for Pharmacoeconomics and Outcomes Research and journals like “Pharmacoeconomics” and “Value in Health”. These advancements are indicative of an increasing realization of the important role played by economic evaluations in healthcare decision-making.

## Economic evaluation techniques

Economic evaluations compare the costs and outcomes of different healthcare services to determine which provide the best value for money. The primary techniques employed encompass (2):

- Cost-Benefit Analysis (CBA): This framework monetizes the cost and benefits of different interventions, making it easier for policymakers to understand the financial return on investment. For every euro or dollar invested, the return is expressed in euro or dollar, for instance. CBA is often considered more intuitive for decision-makers because it directly relates to financial outcomes.
- Cost-Effectiveness Analysis (CEA): CEA evaluates interventions based on their costs relative to their health outcomes, typically measured in natural units such as life years gained or symptoms reduced. This method is particularly useful for comparing interventions that have similar goals but different costs and effectiveness profiles.
- Cost-Utility Analysis (CUA): CUA uses a generic index of morbidity and mortality, such as QALYs or DALYs, to compare the value of different interventions. This approach allows for a standardized comparison across diverse health conditions.

The Incremental Cost-Effectiveness Ratio (ICER) is a key metric in these analyses, representing the additional cost per additional unit of health benefit (e.g., cost per QALY gained) when comparing two interventions (2). This metric is crucial in determining whether an intervention is considered cost-effective within a given context. In addition, the cost-effectiveness plane is often used to visualize these comparisons, plotting cost and effect pairs of different interventions relative to each other (7).

## Current evidence on cost-effectiveness of digital interventions

Research on the cost-effectiveness of digital interventions, particularly for mental health disorders like depression and anxiety, has produced promising results. Numerous systematic reviews have demonstrated that guided internet interventions for depression and anxiety appear to be cost-effective compared to controls (8–13). However, Kolovos et al. (14) found in an individual participant data meta-analysis that internet interventions for the treatment of depression were not considered cost-effective. The evidence for digital interventions in treating substance use disorders is similarly mixed but generally positive. Buntrock et al. (15) reviewed 11 studies and found that the likelihood of internet interventions being cost-effective in this area appears promising. This suggests that digital interventions have the potential to address a wide range of mental health issues, extending beyond depression and anxiety. Despite this potential, interventions aimed at the same condition often differ in their underlying principles, content, and the type and extent of support offered. It is important to consider that these variations may influence treatment outcomes and raise questions about whether evidence from similar interventions can be effectively combined to create comprehensive recommendations regarding their cost-effectiveness.

## Common misconceptions

There are several misconceptions when interpreting results of economic evaluations. A prevalent misconception is that “cost-effective interventions” are synonymous with “being cheap” or “saving money”. Cost-effectiveness measures the value of an intervention by comparing the health outcomes achieved per unit of cost, rather than just the total cost. It assesses the relative costs and benefits of different interventions to determine which offers the best health outcomes for the money spent. An intervention is deemed cost-effective if it provides a good balance between the costs incurred and the health benefits gained, typically measured in quality-adjusted life years (QALYs) or disability-adjusted life years (DALYs) (2). An intervention could be more expensive but still cost-effective if it provides significantly better health outcomes compared to alternatives. For example, a digital mental health intervention may have higher upfront costs due to development and implementation but can be cost-effective by reducing long-term healthcare costs through improved health outcomes and increased productivity.

Another misconception is that if an intervention is cost-effective, it should automatically be funded by payers. This overlooks the complexity of healthcare budgeting and the various factors influencing funding decisions (16). While cost-effectiveness is an important criterion, it is not the sole determinant in funding decisions. Payers must also consider budget impact, affordability, equity, and overall healthcare priorities. An intervention deemed cost-effective may still face funding challenges if the total costs are high or if there are competing priorities for limited resources (16). For instance, a digital intervention for mental health may be cost-effective, but if the initial investment required is substantial, payers might prioritize other interventions that align more closely with immediate healthcare needs or policy goals. Thus, cost-effectiveness must be balanced with practical considerations of budget constraints and strategic priorities. What is considered valuable can vary depending on the perspective – whether societal, healthcare provider, or patient. An intervention that is cost-effective from a societal perspective might not be budget-neutral for a healthcare provider or affordable for patients. This discrepancy can lead to challenges in funding decisions, where the broader benefits of an intervention are recognized, but the immediate financial burden falls on specific stakeholders.

A third misconception, which is related to the second one, is that cost-effectiveness analysis is the best and only metric for making decisions about resource allocations in healthcare. This perspective can lead to an over-reliance on economic evaluations at the expense of other important factors. While cost-effectiveness is a valuable tool for informing healthcare decisions, it should not be the sole criterion (2, 17). Other factors, such as clinical efficacy, patient preferences, ethical considerations, and societal values, play crucial roles in decision-making (18). Healthcare decisions often involve trade-offs that go beyond economic metrics. For example, a digital intervention might be cost-effective but pose ethical dilemmas or be less acceptable to patients. Decision-makers must consider a holistic view that integrates economic evaluations with broader healthcare objectives. Economic evaluations should be part of a broader decision-making framework that balances these various factors. For example, a digital intervention that is highly cost-effective might not be equitable if it disproportionately benefits certain populations over others.

## Discussion and future directions

Digital interventions hold significant potential for improving mental health outcomes in a cost-effective way. However, drawing firm conclusions about their value requires careful consideration of methodological differences and potential biases in studies, as well as the broader context of healthcare decision-making. When designing economic evaluations, several key factors should be considered. These include the objectives of the analysis, the intended audience, the type of economic evaluation to be performed (e.g., cost-effectiveness or cost-benefit analysis), the perspective of the analysis (e.g., societal vs. healthcare), the definitions of the interventions, the target population, the comparators, the time horizon, the analysis plan, the required data types, and the methods of data collection (19). Advancing the field of economic evaluations for digital health interventions requires several key steps.

Firstly, methodological variability in clinical studies plays a significant role. Studies included in the systematic reviews differ in the length of follow-up, the inclusion of development costs, and the choice of comparators, with waitlists and usual care as the most common comparators. However, the distinctions between waitlists and usual care were not always clearly defined (8–15). These differences can significantly affect the results and their interpretation, particularly concerning whether a digital intervention is deemed cost-effective as interventions compared against waitlist controls might appear more effective than when compared against active treatments (20). In this context, a decision-analytic model indicated that digital interventions for generalized anxiety disorder yielded a lower net monetary benefit compared to medication and face-to-face therapy, while offering a greater net monetary benefit than non-therapeutic controls and the absence of any intervention (21). In addition, it is important to consider long-term outcomes in our evaluations. Short-term studies may miss the full range of benefits and costs associated with digital interventions. Evaluating their long-term cost-effectiveness will provide a more comprehensive understanding of their impact on healthcare systems over time. By developing consistent methodologies, particularly in how we define and include cost components, such as whether to account for the development costs of the digital interventions or not, we can achieve more comparable and reliable results across different studies. Secondly, publication bias can skew the overall perception of cost-effectiveness (22). We must encourage the publication of all study results, regardless of their outcomes. Once planned, economic analyses should be conducted regardless of the results on clinical effectiveness. This approach will help us build a more accurate understanding of the true effectiveness and cost-effectiveness of digital interventions for the prevention and treatment of mental health disorders, avoiding the skewed perspectives that can arise from only reporting positive findings. This bias also needs to be accounted for in systematic reviews and meta-analyses to ensure that conclusions reflect the true state of the evidence. Third, the generalizability of study findings is another critical factor. Most research on cost-effectiveness of digital interventions is conducted in high-income countries, which limits the applicability of findings to low-resource settings. Internet access and digital literacy can vary widely between countries, affecting the feasibility and effectiveness of digital interventions in different contexts. Thus, expanding research to diverse settings is vital. Conducting studies in low- and middle-income countries, as well as among diverse populations, will ensure that our findings are more generalizable. This will help to determine whether digital interventions can be effectively implemented on a global scale. Fourth, we must incorporate patient perspectives into our evaluations. By considering the preferences and experiences of the people, who will actually use these interventions, we can ensure that they are not only cost-effective but also acceptable, feasible, and aligned with patient needs. Finally, integrating digital interventions with existing healthcare systems is a critical area for research. Assessing how these interventions can be seamlessly incorporated into current systems will offer valuable insights into their scalability and sustainability, ensuring they can be effectively maintained and expanded within the broader healthcare framework.

## Conclusion

While digital interventions hold significant potential for improving mental health outcomes in a cost-effective manner, careful consideration of methodological differences, potential biases, and broader healthcare objectives is crucial. By addressing these factors, stakeholders can make more informed decisions that balance economic considerations with the overall goal of providing accessible, effective, and equitable mental health care interventions.
